# Prevalence of Patients With Pain Consulting Physiotherapists (PHYSIODOL Survey). A Nationwide Survey in France

**DOI:** 10.1002/ejp.70243

**Published:** 2026-03-03

**Authors:** Thibaut Mussigmann, Jean‐Pascal Lefaucheur, Benjamin Bardel, Arnaud Choplin, Luis Garcia‐Larrea, Charles Quesada

**Affiliations:** ^1^ UR 4391, ENT Team, Faculty of Health Paris‐Est Créteil University Créteil France; ^2^ University Institute of Physiotherapy (IUK), Paris‐Est Créteil University Fontainebleau France; ^3^ Clinical Neurophysiology Unit Henri‐Mondor Hospital, Assistance Publique—Hôpitaux de Paris Créteil France; ^4^ Université Côte d'Azur Nice France; ^5^ NeuroPain Lab Lyon Centre for Neuroscience Inserm U1028 Lyon France; ^6^ NeuroPain Lab Inserm U1028 Jean Monnet University Saint‐Etienne Saint‐Étienne France

**Keywords:** epidemiology, pain, physiotherapy, prevalence: chronic pain, rehabilitation

## Abstract

**Background:**

Pain, whether as a symptom or a chronic disease, is the leading cause of medical consultations but there is no data on whether this is also the case in the practice of physiotherapy.

**Objective:**

To assess the prevalence of patients with pain consulting a physiotherapist in France.

**Methods:**

A questionnaire to be completed online was sent to physiotherapists practicing in France, asking them about the presence of pain in the patients they treated in the previous five days. The questionnaire was sent through representative healthcare organisations and professional societies, from January to September 2024.

**Results:**

A total of 845 questionnaires were completed by physiotherapists, representing 52,497 patient consultations. Overall, 58.7% of patients seen by physiotherapists presented with pain, and pain was the main reason for consultation in 39.5% of patients. Physiotherapists reported a higher proportion of patients with chronic pain than with acute pain. Differences in pain characteristics were observed according to professional practice context: self‐employed physiotherapists more frequently reported pain located in the head or trunk and mixed acute and chronic pain profiles, whereas salaried physiotherapists more often reported acute pain and lower limb pain.

**Conclusion:**

Pain is the major reason for physiotherapy consultations, and chronic pain is commonly encountered. Differences observed between self‐employed and salaried physiotherapists likely reflect variations in care contexts, referral pathways, and predominant fields of clinical activity. These results should inform health authorities of the role of physiotherapists in pain management and promote the development of standardised undergraduate and postgraduate pain education programs.

**Significance Statement:**

This is the first nationwide and representative study to assess the prevalence and characteristics of pain among patients consulting physiotherapists. The results demonstrate that pain, particularly chronic pain, is a major reason for physiotherapy consultations in France. The evaluation of current educational programs and the implementation of standardised pain education for physiotherapists should now be undertaken to ensure best practices and ultimately improve patient outcomes.

## 1 Introduction

The International Association for the Study of Pain defines pain as ‘an unpleasant sensory and emotional experience associated with, or resembling that associated with, actual or potential tissue damage’ (Raja et al. 2020). Pain is a heterogeneous condition that can be a symptom, a medical condition, or a disease in itself (Raffaeli and Arnaudo 2017; Treede et al. 2019). With a prevalence estimated at around 20% (Breivik et al. 2006) and a substantial economic burden across countries (Henschke et al. 2015), pain represents a major public health issue. It is one of the leading reasons for medical consultations worldwide. It accounts for 40% of physician visits in Finland (Mäntyselkä et al. 2001), 39% in Barcelona (Spain) (Calsina‐Berna et al. 2011), 42.5% in Bochum (Germany) (Friessem et al. 2009), and 30% of consultations in Sweden (Hasselström et al. 2002). Across clinics, 50.4% of consultations in Grampian (United Kingdom) are related to pain (Elliott et al. 1999), and overall fluctuates between 5.5% and 30% in Asia, Africa, America, and Europe (Gureje et al. 1998). In France, pain is one of the leading reasons for emergency visits and consultations with physicians (Maisonneuve 2017; Milojevic et al. 2007; Tajfel et al. 2002).

Other health professionals, such as physiotherapists, also play a crucial role in treatment, follow‐up, and disease prevention. There are approximately 2 million physiotherapists worldwide, and the World Confederation for Physical Therapy defined their role as one which ‘provides services that develop, maintain and restore people's maximum movement and functional ability’. While this definition is broad, physiotherapists have also been shown to play a key role in pain management (Ginnerup‐Nielsen et al. 2016). In most countries, physiotherapists receive patients through physician referrals, although some healthcare systems allow direct access. Since referral from physicians is the major patient pathway in France, and pain represents a major proportion of medical consultations, it is expected that a significant proportion of patients treated by physiotherapists seek care for pain, whether as a symptom or as a chronic condition. However, to date, no studies have investigated the proportion of physiotherapy consultations related to pain in any country. Such data could provide valuable insights into the role of physiotherapists in pain management and inform public health authorities.

The first aim of our study was to assess the prevalence of pain patients consulting physiotherapists in France and the prevalence of patients whose pain was the primary reason for consultation. We also collected data on sex, pain characteristics (i.e., acute or chronic), pain site, and pain intensity. Our second aim was to describe the differences between pain prevalence, and pain characteristics, depending on the physiotherapist activity (self‐employee or working in institution). This secondary analysis was designed as a descriptive, system‐oriented approach to explore potential differences in pain‐related consultations across organisational practice settings.

## 2 Methods

The Physiodol project (ethics approval no. 2023‐06‐15‐002) consisted of an online questionnaire sent to physiotherapists in France, asking them about their last 5 days of practice.

### 2.1 Distribution

The online questionnaire was posted on the online platform of the Claude Bernard Lyon‐1 University and was available from January 2024 until September 2024 via the French Society of Evaluation and Treatment of Pain (SFETD) and the French Society of Physiotherapy (SFP). It was also communicated through official physiotherapy institutions in each department of France (Conseil Départemental de l'Ordre des Masseurs‐Kinésithérapeutes, CDOMK) and the regional representative authorities of physiotherapists (Union Régionale des Professionnels de Santé des Masseurs‐Kinésithérapeutes, URPS‐MK). The questionnaire could be sent by email, included in a press release, or posted on the organisations' websites. All physiotherapists actively practicing in France were eligible to participate.

### 2.2 Description

The questionnaire was developed by a multidisciplinary team from two research centers specialising in pain research. The questionnaire was designed to be simple and concise (completion time below 4 min) to maximise the response rate. Pre‐tests were conducted on a small sample of 154 physiotherapists to ensure clarity and accurate understanding. The final questionnaire consisted of two parts. ‘Part 1: Clinical Practice Data’ included eight questions. Six questions focused on the previous 5 days of practice, covering (i) the total number of patients seen; (ii) the number of patients with pain; (iii) the number of patients whose primary reason for consultation was pain; (iv) whether the physiotherapist mainly treated acute or chronic pain, (v) the main pain location profile among patients with pain, and (vi) pain intensity categorised by the physiotherapist based on numeric rating scale: mild (0–3), moderate (4–6), severe (7–10). Two additional questions addressed pain assessment methods, specifically the tools used and the frequency of pain evaluation in daily practice, which reflects usual clinical practice among all physiotherapists. Overall, a patient was classified as having pain if pain was present during consultation, irrespective of intensity or functional impact, and whether or not it constituted the primary reason for consultation. For example, a patient with mild chronic back pain consulting primarily for respiratory rehabilitation would be categorised as ‘patient with pain’ but pain would not be recorded as the primary consultation reason. ‘Part 2: Socio‐Demographic Data’ aimed to establish a general profile of the respondents. The following data were collected: sex, years of experience, type of practice (e.g., self‐employed or salaried), region of practice, and predominant area of practice, which was assessed by asking respondents to select one main field of physiotherapy from a predefined list of 10 options. They were also asked whether they had received specific training in pain management. Physiotherapists were free to skip any question. Data were included in the analysis when the response rate for a given question exceeded 90%.

### 2.3 Analysis

We assessed the representativeness of our results compared to the results from the ‘Demographic Observatory of the National Council of the Physiotherapy’ of 2024, which estimated a total of 105,658 physiotherapists in France, 85% self‐employed and 15% salaried, of whom 52.2% were women (Quesnot et al. 2024). Consequently, for a sample size of 845 responses, the maximum expected margin of error for proportions was 3.36% at a 95% confidence level.

Continuous variables were summarised using the mean and standard deviation (SD). The mean percentage of patients with pain and the mean percentage of patients whose pain was the primary reason of consultation were compared between groups using the Wilcoxon rank‐sum test for non‐normally distributed data. Categorical variables, including pain location, pain type, and pain intensity, were compared using the Chi‐squared test. When the Chi‐squared test was significant, post hoc pairwise comparisons were performed using Fisher's exact test. A statistical significance threshold of *p* < 0.05 was applied for all statistical tests. All analyses were conducted using the statistical software R (version 4.2.0).

## 3 Results

A total of 845 responses were received from physiotherapists, representing 52,497 patient consultations over a 5‐day period.

### 3.1 Description of the Physiotherapists Interviewed

Overall, 660 (82.2%) physiotherapists were self‐employed, 109 (13.6%) were salaried employees, 30 had mixed practice (3.7%), and 4 (0.5%) were in other professional situations.

Self‐employees were predominantly exercising in rheumatology or musculoskeletal field (78.5% of the physiotherapists among 10 domains of practice), and salaried were predominantly exercising in geriatrics, neurology, orthopaedic, paediatric and respiratory medicine (81.8% of physiotherapists among 10 domains of practice).

The sex, field of activity, duration of experience, and the number of different patients seen in the previous 5 days are presented in Table 1. Information on pain‐related training, frequency of pain assessment, and the evaluation method is presented in Table 2.

**TABLE 1:** General Profiles of the physiotherapists interviewed.

Profiles of physiotherapists	All	Salaried	Self‐employed
*Sex*			
Woman	422 (55.4%)	73 (70.2%)	336 (53.8%)
Man	340 (44.6%)	31 (29.8%)	289 (46.2%)
*Predominant area of practice*			
Cardiology	3 (0.4%)	2 (1.8%)	1 (0.2%)
Geriatrics	77 (9.4%)	20 (18.4%)	50 (7.7%)
Neurology	44 (5.4%)	23 (21.1%)	14 (2.2%)
Oncology	32 (3.9%)	10 (9.2%)	20 (3.1%)
Orthopaedics and traumatology	277 (33.7%)	20 (18.4%)	239 (36.9%)
Paediatrics	29 (3.5%)	15 (13.8%)	11 (1.7%)
Respiratory medicine	18 (2.2%)	11 (10.1%)	5 (0.8%)
Rheumatology	299 (36.3%)	6 (5.5%)	269 (41.6%)
Sports Medicine	16 (1.9%)	2 (1.8%)	12 (1.9%)
Urogynecology	28 (3.4%)	0 (0.0%)	26 (4.0%)
Duration of practice (years)	18.31 ± 11.46	15.07 ± 10.75	18.79 ± 11.43
Number of different patients seen in the last 5 days	63.1 ± 32.04	29.86 ± 22.42	68.65 ± 29.70

**TABLE 2:** Profiles and practices related to pain of the physiotherapists interviewed.

Profiles of physiotherapists	All	Salaried	Self‐employed
*Formation related to pain*			
Yes	279 (41.3%)	47 (49.0%)	215 (38.9%)
No	398 (58.8%)	49 (51.0%)	338 (61.1%)
*Frequency of pain assessment*			
Never	2 (0.2%)	0 (0.0%)	2 (0.3%)
Rarely	32 (3.8%)	6 (5.5%)	25 (3.8%)
Often	253 (30.0%)	33 (30.3%)	196 (29.7%)
Always	556 (66.0%)	70 (64.2%)	437 (66.2%)
*Evaluation system*			
None	9 (1.1%)	1 (0.9%)	7 (1.0%)
Numeric rating scale	621 (73.7%)	84 (77.1%)	485 (73.6%)
Without standardised tools	154 (18.3%)	16 (14.7%)	123 (18.6%)
Other^a^	59 (7.0%)	8 (7.3%)	45 (6.8%)

^*a*^ Other refers to various modalities of assessment with standardised tools other than numeric scales, such as body diagrams, quality of life questionnaires or functional scales.

### 3.2 Primary Outcomes (See Table 3)

**TABLE 3:** Pain features reported by the physiotherapists interviewed.

Profiles of physiotherapists	All	Salaried	Self employed	Comparison between salaries and self employed
Patients reporting pain	58.71%	52.52%	59.73%	*p* = 0.02
Pain as the leading cause of consultation	39.50%	20.5%	41.88%	*p* < 0.01
*Main pain intensity*				*p* = 0.07
Mild	86 (10.2%)	15 (13.9%)	62 (9.4%)	
Moderate	580 (69.1%)	66 (61.1%)	463 (70.3%)	
Strong	35 (4.2%)	9 (8.3%)	24 (3.6%)	
Without predominance^b^	128 (15.2%)	16 (14.8%)	102 (15.5%)	
Not determined^a^	11 (1.3%)	2 (1.9%)	7 (1.1%)	
*Main location of pain*				*p* < 0.01
Head or trunk	291 (34.7%)	22 (21.0%)	245 (37.1%)	*p* < 0.01
Upper limb	99 (11.8%)	8 (7.6%)	82 (12.4%)	*p* = 0.19
Lower limb	77 (9.2%)	29 (27.6%)	39 (5.9%)	*p* < 0.01
Not determined^a^	18 (2.14%)	4 (3.8%)	14 (2.1%)	*p* = 0.29
Without predominance^b^	354 (42.18%)	42 (40.0%)	280 (42.4%)	*p* = 0.67
*Main type of pain*				*p* = 0.01
Acute	180 (21.4%)	33 (30.6%)	126 (19.7%)	*p* = 0.01
Chronic	407 (48.4%)	52 (48.2%)	330 (51.6%)	*p* = 0.76
Without predominance^b^	217 (25.8%)	17 (15.7%)	181 (28.3%)	*p* = 0.01
Not determined^a^	37 (4.4%)	6 (5.6%)	3 (0.5%)	*p* = 0.28

^a^ Not determined indicates that no predominant pain intensity, pain location, or pain type could be identified.

^b^ Without predominance indicates that no single category clearly dominated over the others.

#### 3.2.1 Prevalence and Characteristics of Pain Patients Consulting Physiotherapists

Overall, 58.7% of patients seen by physiotherapists during the 5‐day window covered by the questionnaire had pain, and pain was the primary reason for consultation in 39.5% of all patients. Overall, pain was mostly moderate (69.1%). The main location of pain was without predominance (42.18%) or at the head or body trunk (34.7%), and pain was predominantly chronic (48.4%) rather than acute (21.4%) or without predominance (25.8%).

### 3.3 Secondary Outcome (See Table 3)

#### 3.3.1 Differences in Pain Prevalence and Characteristics Between Self‐Employed and Salaried Physiotherapists

The percentage of patients with pain (59.7% vs. 52.5%, *p* = 0.02) and the proportion of patients in whom pain was the leading cause of consultation (41.9% vs. 20.5%; *p* < 0.01) were significantly higher among self‐employed practitioners compared to salaried.

Self‐employed physiotherapists reported more patients with pain located in the head or trunk (37.1% vs. 21.0%, *p* < 0.01) and more frequently a combination of acute and chronic pain without predominance (28.3% vs. 15.7%, *p* = 0.01).

Salaried physiotherapists, on the other hand, reported more patients presenting with acute pain (30.6% vs. 19.7%, *p* = 0.01) and more cases of lower limb pain (27.6% vs. 9.5%, *p* < 0.01).

### 3.4 Exploratory Outcomes

Rheumatology (64.0%) and musculoskeletal‐oriented practice (61.2%) were the predominant area of practice in which pain was most frequently reported. In contrast, lower proportions of patients presenting with pain were observed in paediatric (31.8%) and respiratory physiotherapy (43.2%).

## 4 Discussion

To our knowledge, this is the first large‐scale and representative study to assess the prevalence of pain among patients consulting a physiotherapist. With more than half of the patients reporting pain, these findings empirically corroborate a long‐standing assumption in this field that ‘pain is the most common reason people seek physiotherapy care’ (Jones and Hush 2011).

Chronic pain was the most common type of pain encountered in physiotherapy. This may be explained by the fact that chronic pain often responds poorly to pharmacological treatment, leading physicians to refer these patients to physiotherapists (Marcianò et al. 2023). While reducing pain intensity remains an important objective, the focus of pain management has progressively shifted toward improving patients' quality of life and functional abilities (Scemama et al. 2023). These outcomes are adequately targeted through non‐pharmacological approaches such as exercise (Geneen et al. 2017), cognitive training (Williams et al. 2020), or education (Louw et al. 2016). Non‐pharmacological therapies are almost systematically included in chronic pain recommendations (Ginnerup‐Nielsen et al. 2016; Kligler et al. 2018), and ranked as top priority in pain research for the next years in Europe (Pickering et al. 2025). The increasing importance of non‐pharmacological treatment in pain management, along with the high prevalence of pain patients consulting a physiotherapist, highlights the significant contribution of physiotherapists in the management and follow‐up of chronic pain patients.

Delivering these interventions effectively requires an in‐depth understanding of pain mechanisms, pain modulators, and the biopsychosocial factors influencing pain in order to perform comprehensive assessments and individualise physiotherapy interventions. This underscores the necessity for physiotherapists to maintain up‐to‐date knowledge and skills in pain science and non‐pharmacological management strategies both at undergraduate and postgraduate levels. It is especially important because the pain field has evolved considerably, illustrated by the update of the pain definition in 2020 (Raja et al. 2020), the recent introduction of nociplastic pain (Kosek et al. 2016), or concepts such as central sensitisation and contextual effects, which have become essential components of contemporary pain understanding. Pain science education has also gained increasing importance in clinical guidelines for chronic pain management (Haute Autorité de Santé 2019, 2023) and is considered a core competency for physiotherapists while its effective implementation requires specific, advanced knowledge and skills.

Albeit French physiotherapy undergraduate training underwent a major reform in 2015, there is no standardised curriculum in pain education. The only study conducted in 2018 on the actual pain program in France showed that undergraduate training remains insufficient and does not follow international guidelines (Osinski and Barde‐Cabusson 2018). Similar findings have been reported in Europe (Skidmore et al. 2025) and in other countries (Hoeger Bement and Sluka 2015; Venturine et al. 2018; Wideman et al. 2020). This issue likely extends beyond physiotherapy to other allied health professions such as psychology, nursing (Skidmore et al. 2025), occupational therapy (Reyes and Brown 2016), as well as speech or psychomotor therapy, for which no data are currently available to our knowledge. Insufficient pain education has also been documented in medical curricula (Briggs et al. 2015; Mezei et al. 2011; Shipton et al. 2018), which may have important consequences, as physicians act as gatekeepers and referral agents within healthcare systems. In this context, alignment with the European Federation for pain (EFIC) and IASP curricula would allow for a better integration of pain education (Hoeger Bement et al. 2014; Shipton et al. 2023), which already serve as foundation for consensus on undergraduate curriculum for physiotherapists across different countries (Augeard et al. 2022; Hoeger Bement et al. 2014; Hush et al. 2018; Reezigt et al. 2024) as well as for other allied health professions (Van Lankveld et al. 2020). Innovative learning strategies, such as Objective Structured Clinical Examination (OSCE) (Barreveld et al. 2021) or Massive Open Online Courses (MOOC) (Bettiol et al. 2022) would be perspectives to improve the undergraduate pain education in physiotherapy.

Regarding postgraduate pain education, 41.3% of physiotherapists reported having received continuing education related to pain in our study. Although this may seem encouraging, it should be interpreted cautiously, as any mention of pain‐related content was counted as positive, regardless of the date or duration of the training. Most of these courses consisted of short continuous education programs (approximately 2 days) completed after graduation, and none of the respondents reported having completed a university‐level program specifically dedicated to pain management for non‐physician health professionals. Since more than half of physiotherapists in France regularly manage patients with pain, there is a need as well for standardised postgraduate training based on international guidelines. This is crucial given the heterogeneity of physiotherapy practice in France (Matharan et al. 2009; Quesnot et al. 2024). Such improvements would be feasible by relying on the international standards already established by the IASP and the EFIC, which also provides a postgraduate certification through the European Diploma in Pain Physiotherapy (https://europeanpainfederation.eu/education/pain‐exams/). These standards have also been incorporated into broader and complementary educational approaches for self‐employed physiotherapists (Van Dijk et al. 2025), which could serve as a model for future postgraduate pain training.

Given that clinical activities and patient profiles vary across professional settings, pain education should also reflect these differences. Accordingly, it was expected that patient profiles would differ between salaried and self‐employed physiotherapists. Importantly, these professional statuses are also associated with different predominant areas of activity, as observed in our results.

Self‐employed encounter a higher proportion of patients with pain, and twice as many cases in which pain was the primary reason for consultation. This is consistent with the predominance of musculoskeletal and rheumatological practice among self‐employed physiotherapists, fields of practice that are inherently associated with a higher burden of pain‐related consultations. The difference in clinical focus may stem from the fact that French salaried physiotherapists often work in hospital or rehabilitation settings, where physiotherapy is delivered as part of a multidisciplinary care plan rather than being primarily centered on pain management, resulting in fewer pain‐focused consultations. In contrast, Self‐employed physiotherapists usually work independently or in small private practices, and patients may consult directly for pain‐related issues: In France, the law of May 19, 2023 established a limited direct‐access mechanism enabling patients to consult physiotherapists without prior medical prescription for specific conditions, namely acute low back pain, ankle sprains, and knee sprains. This applies predominantly to private practice settings (i.e., mainly self‐employed), remains subject to specific eligibility criteria (Ministère de la Santé et de la Prévention 2023) and this controlled direct‐access is currently being gradually expanded (Ministère des Solidarités et de la Santé 2025). Additionally, self‐employed physiotherapists may be more prone to maintain a long‐term therapeutic relationship with patients who are no longer hospitalised but still require regular follow‐up to support adherence in non‐pharmacological treatment and manage fluctuations in their condition.

Furthermore, salaried physiotherapists reported more patients presenting with acute pain, lower limb pain location, and their predominant area of practice tended to focus on geriatrics, orthopaedics, neurology, paediatrics, and respiratory medicine. It is possible that, in hospital and rehabilitation settings, patients who see physiotherapists often present with multiple comorbidities, postoperative recovery, or conditions such as stroke, asthma, or chronic obstructive pulmonary disease, where pain is less prevalent or not the main reason for physiotherapy. Pain may also be underreported or underestimated in these populations, particularly among geriatric and paediatric patients, where assessment remains challenging (Afenigus 2024).

Taking altogether, these differences between the type of activity should be interpreted as reflecting variations in care contexts, referral pathways, and patient trajectories.

Self‐employed physiotherapists more frequently encounter pain located in the head or trunk, as well as mixed acute and chronic pain without a predominant type, and their main area of practice tended to focus on rheumatology and musculoskeletal disorders. They may be more likely to treat patients with cervical or low back pain, one of the most prevalent conditions in general practice (St. Sauver et al. 2013) that would be redirected to self‐employed physiotherapists (Peurois et al. 2023) and individuals seeking advice or support for mild–moderate functional complaints. Previous research on self‐employed physiotherapists confirms that rheumatology and musculoskeletal disorders represent their main fields of area (Panchout et al. 2017). However, no additional information is available regarding the diagnosis of the patients that see a physiotherapist.

The proportion of patients with chronic pain did not differ between self‐employed and salaried physiotherapists. However, no detailed information is available regarding the specific diagnoses of patients consulting physiotherapists.

Our findings reported that 42% of the patients who consult a physiotherapist do not have pain. Indeed, physiotherapists also promote rehabilitation therapy related to motor, cognitive, speech, respiratory, or vestibular disorders that do not necessarily imply the presence of pain.

This study has several limitations that should be considered. With 845 respondents among approximately 105,658 practicing physiotherapists in France, the response rate was 0.8%, which may limit generalisability. This limited response likely reflects the absence of financial incentives and the indirect dissemination strategy through professional organisations. Despite these limitations, the distribution of sex, type of practice, and sex within each practice category among respondents was comparable to national physiotherapist demographics in France. Our survey asked physiotherapists to recall retrospectively their activities over the last 5 days. It is possible that they were not as precise as they would have been if we had collected data prospectively. In the initial drafts of this survey, we considered asking physiotherapists to prospectively report their activities every day, but this resulted in a significant reduction in the response rate. Therefore, we opted for this retrospective study over a short period of a few days, although the choice of only 5 days was arbitrary and not necessarily representative of the entire annual activity. Another limitation relates to the duration of the recording period. Indeed, consultations for pain may vary throughout the year, while we only asked about the number of patients with pain during a single 5‐day window within the year. Also, a potential selection bias related to the online distribution methodology cannot be excluded. Physiotherapists with limited digital literacy may have been underrepresented, or physiotherapists with a greater interest or sensitivity to pain management may have been more likely to respond.

From an educational perspective, these findings support the need for standardised undergraduate pain curricula aligned with international IASP and EFIC recommendations. In addition, structured and recognised postgraduate pain education programs appear necessary to complement short continuing education courses and to address pain management across various clinical contexts. Achieving these changes requires coordinated involvement of universities and training institutes, health authorities, professional bodies, and scientific societies.

Finally, this study did not account for the evaluation of care‐induced pain, a situation not limited to invasive procedures but also present during contact or exercise (Cornec et al. 2025; Smith et al. 2019), but this would be relevant for future research to ensure the improvement of good practices when dealing with a patient with pain. It also raises the issue of establishing a consensus on acceptable pain levels for maintaining physiotherapy sessions, especially in the context of chronic pain (Smith et al. 2020).

## 5 Conclusion

This nationwide study provides the first empirical evidence that pain represents a major component of physiotherapy practice in France, with nearly three out of five patients presenting with pain and two out of five consulting primarily for this reason. Compared with the estimated 42% prevalence of chronic pain in the French population (Observatoire Français de la Douleur et des Antalgiques 2025), the higher proportion observed in physiotherapy consultations likely reflects both referral pathways and the role of physiotherapy in the management of chronic pain. The predominance of chronic pain underscores the essential role of physiotherapists in delivering non‐pharmacological interventions within contemporary multimodal pain management. Importantly, the differences observed between self‐employed and salaried physiotherapists should be interpreted as reflecting distinct combinations of organisational care settings, referral pathways, and predominant fields of clinical activity, which together shape patient profiles and trajectories, rather than differences in professional competence. These contextual differences warrant tailored educational strategies that account for the diversity of physiotherapy practice. Overall, these findings highlight the need for strengthened pain education aligned with international IASP and EFIC standards to ensure physiotherapists possess the contemporary knowledge and competencies required to optimise patient outcomes. They may also inform public health policies, particularly with regard to the training of healthcare professionals and the development of awareness initiatives targeting those who frequently encounter patients with pain.

Future studies should specifically investigate pain prevalence and characteristics across major physiotherapy disciplines.

## Author Contributions

T.M.: conceptualisation, methodology, investigation, analysis, writing, supervision. J.‐P.L.: conceptualisation, methodology, analysis, writing. B.B.: conceptualisation, methodology, analysis, writing. A.C.: conceptualisation, methodology, analysis, writing. L.G.‐L.: conceptualisation, methodology, analysis, writing. C.Q.: conceptualisation, methodology, investigation, analysis, writing, supervision.

## Funding

The authors have nothing to report.

## Conflicts of Interest

The authors declare no conflicts of interest.
